# Abortion in Cows**Read before the Farmer’s Club March* 13, 1883.

**Published:** 1883-04

**Authors:** Ehrick Parmly


### Art. XII.—ABORTION OF COWS.*

BY EHRICK PARMLY, M.D.

I find the cause of abortion so well given in Law's book on Veterinary Medicine that I copy the paragraph:

"Blows or pressure on the abdomen, slips, falls, riding of animals in heat, diseases of the abdominal organs (tympanitis from wet, frosted or musty fodder, inflammation of the bowels, diarrhœa, poisoning by irritants taken with the food or otherwise, venal calculi or other diseases of the kidneys or bladder), stalls too much inclined backward, overfeeding, plethora, hot, damp, relaxing stables,severe muscular exertion after long rest, exhausting feeding for milk at the expense of the system, breeding at too early an age, proximity to or contact with slaughter houses or dead and decomposing animal matter, especially the abortion discharges of other animals, drinking putrid or iced water, disease, deformity or death of the fœtus, feeding on ergoted grasses or smelty wheat and corn, and, finally, the presence in the passages of a microscopic vegetable parasite (leptothrix vaginalis) which is easily transferred from one animal to another so as to procure abortion."

In the above I find little to aid me in fixing upon the cause or causes of a run of abortion in my herd.

A short history of the cases may throw some light on the subject. The marked feature is the first abortion which was a strange cow brought and introduced into the herd early in August. She aborted Aug. 25th in the field with the herd. She was 5 months gone in pregnancy. I then attributed it to the hard reception she received from several of the herd. The next case occurred Dec. 5th at 7 months 29 days.

| | | | | | | |
|---|---|---|---|---|---|---|
| The 3d | Dec. | 20th, | at 6 | months | 2 | days. |
| " 4th | Jan. | 4th, | at 5 | " | 18 | " |
| " 5th | " | 6th, | at 5 | " | | |
| " 6th | " | 20th, | at 6 | " | 17 | days. |
| " 7th | " | 29th, | at 6 | " | 23 | " |
| " 8th | Feb. | 20th, | at 6 | " | 3 | " |
| " 9th | " | 22d, | at 7 | " | 10 | " |
| " 10th | " | 27th, | at 3 | " | 25 | " |

**Read before the Farmer's Club March* 13, 1883.

The third case I attributed to fighting, and gave orders to not allow the fighting cows to be with the others when out for exercise. The fourth case caused me strongly to suspect that I had an infectious form of disease in the herd, and I at once isolated the animals as far as possible to do so, and made some changes in feeding, but with apparently very little benefit, as I have since had 5 cases. They are gradually coming nearer their term before aborting, which looks favorable.

I throw out of question the food they are receiving as No. 1, 2 and 3, aborted on pasture and no feed. The others had good hay and corn stalks, and either a light feed of corn meal and bran, or a still lighter feed of cotton seed meal. All that was fed was in good condition so far as I am able to judge.

I find from several writers on the subject that abortions increase in frequency as pregnancy progresses, being greatest the sixth, seventh and eighth month, and most frequent in December, January and February. Their advice is to raise your cows rather than buy them, and thus avoid one result, *i. e.*, the introduction of disease into your herd from outside. The excitement attending the removal, by rail or otherwise, of pregnant cows increases the tendency to abort. Breeding too young, and milking too long in pregnancy is objectionable.

The cow aborts more frequently than any other domestic animal. In respect to her reproduction she is in a more artificial and trying condition than any other animal, as often she is given little or no rest, and is called upon to both give milk and nourish a fœtus, thus having two opposing forces at work at the same time.

In my herd the aborting animals cleaned readily. The fœtus, when old enough, was alive, and in one case, at three months and twenty-five days, the fœtus and membranes came away together showing a relaxed condition, and death probably began at the placenta. In some herds a different state of things is shown. Death begins with the fœtus, and when it is thrown off it has evidently been dead some time and is cast out as a foreign body, and the afterbirth is a long time in passing. There are evidently two different germs at work, whatever they may be.

Regarding this as an endemic and infectious the practical question comes up, what to do in the way of treatment of those manifesting symptoms of impending abortion, and of prevention for those still in apparent health.

Isolation is often not practicable when all buildings are well filled. Temporary ones it takes time to put up, and just here it would seem no more than simple prudence for every one having a herd of any value to have on his farm a few inexpensive buildings separated from the principal barn for a hospital or quarantine, or lying-in asylum. The daily or at least weekly removal of all manure from its yards would be advisable at all seasons when there is any sickness.

I hope some information and suggestions from the Farmer's Club will be given that will enable those meeting with losses to fight successfully this very destructive disease, and if not, it will be the first time I ever came before this body without receiving information of value.

Treatment, so far as I can learn, consists in isolating at once all animals that have aborted, or that show symptoms of aborting.

Examine food for each, and make any change that is indicated.

Disinfect the stables and observe the strictest cleanliness.

Allow no accumulations in the yard, not even for a day.

The man who attends the diseased animals should not come in contact with the well ones.

When a cow has aborted, remove by the hand the afterbirth, bury or burn at once, and cleanse the womb by the use of a syringe. Various things are recommended for this purpose, carbolic acid, parmayanate of potassum.

The animal that has aborted should not be bred until several periods of heat has passed. Some fix the date proper for breeding again as after the time she should have come in. Others say always allow six months rest, irrespective of the time of aborting. Others say it is difficult to get a cow in calf if she is allowed too long a rest, so lose no time in breeding.

By breeding too soon one incurs the risk of continuing the disease indefinitely. I would prefer the other risk.

The questions that arise in connection with this disease are:

1. Cause or causes? Period of incubation?

2. Can it be produced in a herd starting from the aborting of a perfectly healthy cow resulting from external or mechanical injury?

3. Can it spread from a cow, by her droppings or otherwise, prior to her aborting? In other words can she, prior to her aborting, be a source of infection?

4. Can a cow, not pregnant, carry the disease into another herd, or rather, may an unimpregnated cow have this disease in her system, of which abortion is the most marked or culminating feature, and be able to communicate it to other animals, pregnant or not pregnant?

5. Can the disease be transmitted by the use of a bull from an infected herd, or by a bull that has been recently coupled with cows that have aborted? Some writers are very positive on this point.

6. How long an absence from a diseased herd would be necessary to render it safe to introduce a calf, a bull, or an unimpregnated female in a healthy herd?

7. Has it ever been noticed that the disease is conceivable from the cow to other domestic animals, as, for instance, mares in foal kept in the same building, or breeding sheep or hogs in the same enclosure?

One friend informed me that he had not a live calf for the period of eighteen months, during which time he fed a mixture of rye, oats and corn. The rye was raised on his farm. The oats and corn he bought. They were ground together as used. He was told by a physician to stop the rye, which he did, and in a short time the abortions ceased. This is interesting, as it shows that with the removal the trouble was ended, and that there was nothing of an infectious nature produced.

I heard of another case where 101 out of 132 cows in a herd aborted during the year. No steps were taken to control the disease. It ceased the following year (four years ago). The herd has since been healthy. The owner has no information to give. He is something of a fatalist. Says everyone must expect bad luck at some time. Science will never advance with men of that stamp.

Mr. William Crozier advises to cleanse a cow thoroughly after abortion by introducing the hand, and follow with a vaginal injection of a pail of warm rain water with soft soap and borax. Then follow with a pint of linseed oil, which he says is soothing to the womb. Mr. Todd's remark about the maternal instinct being violated by removing the calf from the mother I do not think has any effect, but the removal of the calf causes the dam to come in heat sooner than she otherwise would, and if bred on this first heat the cow has not sufficient rest. I have known cows deprived of their calves to come in heat on the fifth day after calving.

Mr. Crozier last year had a case of bad presentation. He spent some hours in trying to turn the calf, but did not succeed. He then dismembered the calf, and succeeded in getting it away. The second day after she came in heat, and he had her covered. She became pregnant from this intercourse, and he wrote me a few days ago that she was about to drop a calf at term.

I have bred cows so that their calves were only ten months apart. I will not do it again. Twelve months is better. A cow bred too rapidly must, even with the best care, become prematurely old.

